# The balance between helper T 17 and regulatory T cells in osteoimmunology and relevant research progress on bone tissue engineering

**DOI:** 10.1002/iid3.70011

**Published:** 2024-09-12

**Authors:** Shuyu Zhu, Jing Zhou, Zhigang Xie

**Affiliations:** ^1^ Kunming Medical University School of Stomatology and Affiliated Stomatology Hospital Kunming Yunnan Province China

**Keywords:** bone tissue engineering, cell balance, osteoimmunology, regulatory T cells, T‐helper 17 cells

## Abstract

**Background:**

Bone regeneration is a well‐regulated dynamic process, of which the prominent role of the immune system on bone homeostasis is more and more revealed by recent research. Before fully activation of the bone remodeling cells, the immune system needs to clean up the microenvironment in facilitating the bone repair initiation. Furthermore, this microenvironment must be maintained properly by various mechanisms over the entire bone regeneration process.

**Objective:**

This review aims to summarize the role of the T‐helper 17/Regulatory T cell (Th17/Treg) balance in bone cell remodeling and discuss the relevant progress in bone tissue engineering.

**Results:**

The role of the immune response in the early stages of bone regeneration is crucial, especially the impact of the Th17/Treg balance on osteoclasts, mesenchymal stem cells (MSCs), and osteoblasts activity. By virtue of these knowledge advancements, innovative approaches in bone tissue engineering, such as nano‐structures, hydrogel, and exosomes, are designed to influence the Th17/Treg balance and thereby augment bone repair and regeneration.

**Conclusion:**

Targeting the Th17/Treg balance is a promising innovative strategy for developing new treatments to enhance bone regeneration, thus offering potential breakthroughs in bone injury clinics.

## Graphical abstract

In this review, we summarized the implication of T‐helper 17/Regulatory T cells balance (Th17/Treg) on the dynamics of bone cell remodeling and relevant mechanisms. We also reviewed the current research progress on bone tissue engineering, which integrates new materials and innovative technologies to intervene in Th17/Treg in promoting bone repair and regeneration.

## 1 INTRODUCTION

Inflammation, mechanical force, tumors, and physiological atrophy lead to bone injuries. Without effective treatments, the local lesions may lead to devastating systemic damage.^1^, ^2^, ^3^ Bone injury restoration is always a central issue of orthopedic clinics. Bone regeneration is a well‐regulated dynamic process, involving the activation, resorption, reversal, and formation phases through various signal transduction mechanisms.^4^, ^5^ This process is crucial for maintaining bone homeostasis and facilitating injury healing.^6^, ^7^, ^8^ With the improvement of our osteoimmunological knowledge, the critical role of the immune system in bone metabolism has been more and more emphasized.^9^

This review provides a comprehensive summary of the implication and mechanisms of Th17/Treg on osteoclasts, mesenchymal stem cells (MSCs), and osteoblasts activity. Additionally, bone tissue engineering strategies aiming to modulate Th17/Treg for bone regeneration are also reviewed.

## 2 THE CRUCIAL ROLE OF THE IMMUNE SYSTEM IN THE EARLY STAGES OF BONE REGENERATION

Bone injuries are often accompanied by cellular debris, necrotic tissues, pathogens, and other hazardous substances. Before fully activation of the bone remodeling cells, the immune system needs to clean up the microenvironment in facilitating the bone repair initiation.

In response to infection or injuries, the Pattern Recognition Receptors (PRRs) of the immune cells primarily identify Pathogen‐Associated Molecular Patterns (PAMPs) or Damage‐Associated Molecular Patterns (DAMPs). The recognition triggers a cascade of protein‐based cleavage and activation processes through stimulation of the complement system. Additionally, neutrophils, macrophages, and other innate immune cells are recruited to the site of infection or injury and activated to phagocytize hazardous substances under the control of inflammatory cytokines. The removal of hazardous substances creates an appropriate microenvironment conducive to bone regeneration.^10^, ^11^

Neutrophils are the pioneer innate immune cells to arrive at the site of injury or infection. After recognizing PAMPs and DAMPs by PRRs, neutrophils clean up the site in various ways, such as phagocytosis, and respiratory burst, and produce Reactive Oxygen Species (ROS) to decontaminate harmful substances. Neutrophil Extracellular Traps (NETs) can also trap and confine harmful substances to prevent their spread. However, excessive pro‐inflammatory reactions may exacerbate local tissue damage and lead to disease progression, such as the aggravation of periodontal disease.^12^ It was suggested that neutrophils may functionally polarize to pro‐inflammatory N1 subgroups and anti‐inflammatory N2 subgroups.^13^, ^14^, ^15^ The worsening of periodontitis might be associated with hyperactivity of the N1 subgroup. These pro‐inflammatory neutrophils promote extensive NETs and the secretion of pro‐inflammatory cytokines that exacerbate gingival tissue damage and alveolar bone resorption.^12^

The examples of periodontal disease demonstrate the intricate role of neutrophils, as they seem to act both as defenders against infection and attackers for exacerbation. The interaction between neutrophils and periodontal pathogens can result in immunological imbalance, creating an environment of immune dysregulation that causes the deterioration of periodontitis. Neutrophils exhibit dual functions of protection and destruction in periodontitis, which may also extend to other bone injuries.^16^

Following the arrival of neutrophils, macrophages migrate to the site of injury to phagocytize cellular debris and pathogens and secrete additional inflammatory mediators, thereby further enhancing the inflammatory response and recruiting other immune cells. Recently, Growing studies have been examining the implications of macrophages on the skeletal system. Under the influence of monocyte chemoattractant protein 1 (MCP‐1),^17^ macrophages aggregate and release numerous bioactive substances including tumor necrosis factor α (TNF‐α), interleukin‐1 (IL‐1), transforming growth factor β (TGF‐β), platelet‐derived growth factors, basic fibroblast growth factors, insulin‐like growth factors among others. These cytokines facilitate MSCs differentiation into osteogenic and chondroblastic cells while promoting angiogenesis and tissue repair.^18^, ^19^

Macrophages demonstrate significant plasticity and heterogeneity when stimulated by the surrounding.^20^ M1 macrophages exhibit enhanced antigen‐presenting ability and pro‐inflammatory properties through the release of cytokines such as IL‐23, TNF‐α, and ROS. In contrast, M2 macrophages secrete anti‐inflammatory cytokines and angiogenesis factors to modulate inflammation, initiate tissue repair processes, and ultimately promote osteogenesis.^21^ The transition from M1 to M2 state is key for shifting from a tissue injury state to a tissue repair state.^22^, ^23^ Numerous studies have demonstrated that intervention of M2 macrophages in bone tissue engineering can establish an immune microenvironment conducive to bone repair, representing an effective strategy for promoting bone regeneration.^24^, ^25^, ^26^

To sum up, the immune system primarily functions to aggregate and activate innate immune cells such as the complement system, neutrophils, and macrophages in the early stages of injury. These cells decontaminate infection and cell debris at the site of injury through diverse mechanisms. Additionally, under the control of specific cytokines released from macrophages, MSCs are recruited and promote angiogenesis. Before full activation of the bone remodeling cells and adaptive immune cells, innate immunity has cleaned up the microenvironment and laid the foundation for bone repair initiation.

## 3 THE DOMINANT INFLUENCE OF THE IMMUNE SYSTEM ON THE MICROENVIRONMENT

The significance of the intricate relationship between the bone system and the immune system has long been recognized.^27^ Arron et al. defined osteoimmunology as a discipline that investigates the interaction between bone remodeling cells and immune cells, highlighting the crucial role played by the immune system in bone metabolism.^9^ Following preliminary clearance to the local microenvironment after bone injury, the immune system continues to create favorable conditions for bone regeneration.

T‐helper 17 cells (Th17) are one subset of helper T cells, whose differentiation is facilitated by TGF‐β1, IL‐6, and IL‐23 produced by antigen‐presenting cells.^28^, ^29^ The function of Th17 is regulated by retinoic acid‐related orphan receptor γt (RORγt), a transcriptional regulatory factor.^30^

Sakaguchi et al. were the pioneers in identifying Regulatory T cells (Tregs) in 1995, highlighting their critical function in bone homeostasis.^31^, ^32^ Tregs differentiation occurs in response to IL‐2 and TGF‐β1 while expressing Forkhead Box P3 (Foxp3) as a phenotypic‐specific transcription factor. Foxp3 is currently considered both a specific marker of Tregs and an important molecule involved in activating or inhibiting their function.

Extensive research has been dedicated to investigating the implication of Th17 and Tregs on the skeletal system.^33^, ^34^, ^35^, ^36^, ^37^ For instance, local immune responses play a pivotal role in the pathogenesis and progression of periodontitis‐associated bone resorption, of which Tregs exert protective effects while Th17 exhibits destructive properties.^28^, ^38^, ^39^ The homeostasis of alveolar bone relies on Th17/Treg balance, whereas pathological alveolar bone resorption arises from an imbalance of Th17/Treg activity.^40^, ^41^ This review will elucidate the implication of Th17/Treg on MSCs, osteoblasts, and osteoclasts.

### 3.1 The implication of Th17 on osteoclasts, MSCs, and osteoblasts

Th17 was suggested to be one of the foremost cells influencing immune response and bone homeostasis.^42^, ^43^ Th17 may generate various cytokines, of which IL‐17 is the hallmark product.^44^, ^45^, ^46^

It is widely acknowledged that IL‐17 is a pro‐inflammatory cytokine to promotes osteoclast activity,^47^ which correlates with conditions such as erosive arthritis and periodontitis.^48^, ^49^ The underlying mechanism primarily involves RANKL‐RANK, a pair of signaling molecules that facilitate osteoclastogenesis.^50^

Th17 secreted IL‐17 may induce osteoclast support cells (such as osteoblasts) to secrete RANKL,^51^ thereby increasing the expression of RANK on the surface of osteoclasts and enhancing their responsiveness to RANKL stimulation.^52^ Additionally, Th17 expresses RANKL directly^43^ and upregulates the ratio of RANKL/OPG to promote osteoclastogenesis.^53^ The binding between RANKL and RANK activates intracellular signal transduction molecules, such as tumor necrosis factor receptor‐associated factor 6 (TRAF6). Activation of TRAF6 further initiates a cascade of downstream signaling pathways, including MAPK as well as NF‐κB signaling pathways, which are crucial for osteoclastogenesis.^54^

It is worth noticing that the upstream regulators of IL‐17‐RANKL axis may important for understanding and leveraging this signaling pathway to prevent pathological bone loss. In a psoriasis model, Triggering receptor expressed on myeloid cells 1 (TREM‐1) blockade significantly reduced the number of Th17 cells and subsequently decreased IL‐17 secretion, indicating that TREM‐1 positively regulates Th17 responses.^55^ Furthermore, Bostanci et al. showed that the microinjection of a TREM‐1 blocking peptide (LP17) into a murine ligature‐induced periodontitis model downregulated IL‐17 gene expression significantly, thereby downregulated the RANKL/OPG ratio and suppressing osteoclastogenesis. This mechanism may offer a promising therapeutic strategy for periodontitis.^56^

In addition to the above signaling pathways, researchers have also directed their attention towards the role of IL‐17 in energy metabolism. Specifically, IL‐17 enhances the energy metabolism of macrophages derived from bone marrow and facilitates their differentiation into osteoclasts. This regulatory process is dependent on Glutamine (Glu), and inhibiting Glu effectively impedes IL‐17‐induced osteoclastogenesis.^57^ This mechanism brings us the potential of a novel strategy to cure bone loss in patients suffering from osteoporosis.^58^

Contrary to the well‐established osteoclastogenic implication of IL‐17, IL‐17 may also exert an osteoblastogenic role in bone homeostasis.^53^ Several experiments have shown that IL‐17 may influence the proliferation and differentiation of MSCs into osteoblasts.^59^, ^60^, ^61^, ^62^, ^63^ The underlying mechanisms may involve the generation of ROS and activation of the activator of transcription 1 (Act1).^53^ Additionally, IL‐17 might promote mineralization by enhancing the expression of runt‐related transcription factor 2 (Runx2).^64^ Furthermore, IL‐17 could potentially enhance osteoblast resistance against ferroptosis and facilitate osteoblastogenesis through the interaction of phosphorylated signal transducer and activator of transcription 3 (pSTAT3) with nuclear factor erythroid 2‐related factor 2 (NRF2).^65^

However, in rat models, IL‐17 was not found to contribute to bone formation.^66^ Some researchers propose that differential expression of IL‐17 receptor subtypes across different species may explain the diversity of IL‐17 effects on MSCs and osteoblasts.^53^ Specifically, the IL‐17 receptor family comprises IL‐17RA to IL‐17RE,^67^ with IL‐17RA and IL‐17RC being the mostly high‐expressed subtypes in human MSCs and mouse skull cells, whereas in rat skull cells, the predominant receptor subtypes are IL‐17RB/D/E. Therefore, the osteoblastogenic effect of IL‐17 may be mediated by IL‐17RA/C. Additionally, there is speculation that IL‐17 only exerts an osteoblastogenic role during the early stages of healing.^59^ However, further investigation is required to elucidate specific mechanisms.

In addition to IL‐17, other cytokines secreted by Th17 have pro‐inflammatory capacity.^44^, ^45^, ^68^ Among these cytokines, TNF‐α activates various signaling pathways that exert detrimental impacts on the skeletal system. TNF‐α upregulates RANKL expression in macrophages and B cells, thereby activating and enhancing NF‐κB as well as stress‐activated protein kinase/c‐jun terminal kinase (SAPK/JNK). These two signaling pathways play critical roles in osteoclastogenesis.^69^, ^70^ Simultaneously, TNF‐α enhances the Wnt signaling pathway inhibitor dickkopf‐related protein 1 (DKK1)^71^, ^72^ and increases purinergic receptor p2y (P2Y2) expression to induce apoptosis of osteoblasts.^73^ Above all, Th17 promotes osteoclast activity while inhibiting osteoblast function, leading to an imbalance in bone homeostasis.

### 3.2 The implication of tregs on osteoclasts, MSCs, and osteoblasts

Tregs not only play a role in hampering the differentiation of Th17 by emitting anti‐inflammatory cytokines, but also influence the formation, function, and apoptosis of osteoclasts. With direct interaction and/or secreted cytokines, Tregs inhibit inflammatory response and bone absorption.^37^, ^74^

For instance, Tregs secrete IL‐10 alone or in combination with RANKL secreted by other cells to reduce the expression of nuclear factor of activated T cells cytoplasmic 1 (NFATc1) in macrophages while preventing macrophages from differentiating into osteoclasts^75^ and hindering osteoclast precursor cells (OCPs) from maturing into functional osteoclasts.^76^, ^77^ Moreover, Tregs utilize cytotoxic T‐lymphocyte antigen‐4 (CTLA4) to induce apoptosis of OCPs by binding to CD80/CD86 on OCPs.^37^, ^78^ Other studies have revealed that TGF‐β1 secreted by Tregs may induce osteoclast apoptosis through the Smad pathway while potentially interfering with MAPK and Bcl2 family members.^79^

Additionally, Tregs regulate the function of osteoclasts during inflammation by responding to extracellular stimulation to avoid excessive reactions of osteoclasts.^74^

The mechanisms of Tregs on osteoclasts are intricate. Specifically, TGF‐β1, secreted by Tregs and capable of inducing apoptosis of osteoclasts. However, TGF‐β1 is also synthesized and secreted by osteoclasts themselves, and is able to promote their functional activities.^80^ Studies have indicated that inhibition of TGF‐β signal transduction hampers the formation of the TRAF6‐TAB1‐TAK1 complex. Inhibition of the binding between TRAF6‐TAB1‐TAK1 complex and Smad3 would affect the RANKL‐induced osteoclast signaling pathway.^81^ Therefore, as a significant autocrine factor for RANKL‐induced osteoclast formation, TGF‐β is indispensable.^82^

Scholars have speculated two reasons for underlying mechanisms behind the dual effects of TGF‐β1 on osteoclasts. One is that the bidirectional effect of TGF‐β on osteoclast differentiation might be dependent on Smad3 or Smad1 signal transduction.^83^ It is well‐known that TGF‐β binds to its receptor and activates downstream Smad proteins, which subsequently translocate into the nucleus and regulate related gene expression.^84^ A study revealed that Smad3 and Smad1 signals induce and inhibit RANK expression, respectively.^83^ The others speculated the reason attributed to the concentration of TGF‐β1. Specifically, a minimal amount of TGF‐β1 enhances the differentiation of osteoclasts stimulated by RANKL or TNF‐α, whereas an elevated concentration of TGF‐β1 promotes osteoclasts to secret OPG and then inhibits osteoclasts activity.^85^, ^86^

Tregs also influence on MSCs and osteoblasts functions. By secreting TGF‐β to activate intracellular signals such as Smad‐related proteins as well as MAPK, Tregs induce osteogenic differentiation of MSCs.^87^, ^88^, ^89^, ^90^ Moreover, the PI3K/AKT/mTOR/S6 kinase 1 signaling pathway is involved in promoting the survival and migration of osteoblasts.^91^ Additionally, Tregs may facilitate the assembly of NFAT1‐SMAD3 transcription complex in CD8 + T cells, leading to the expression of Wnt10b to activate the Wnt signal in osteoblasts, thereby promoting bone formation.^92^, ^93^ Furthermore, TGF‐β1 exhibits chondrogenic effects while inhibiting cartilage absorption.^94^

**Figure 1:**
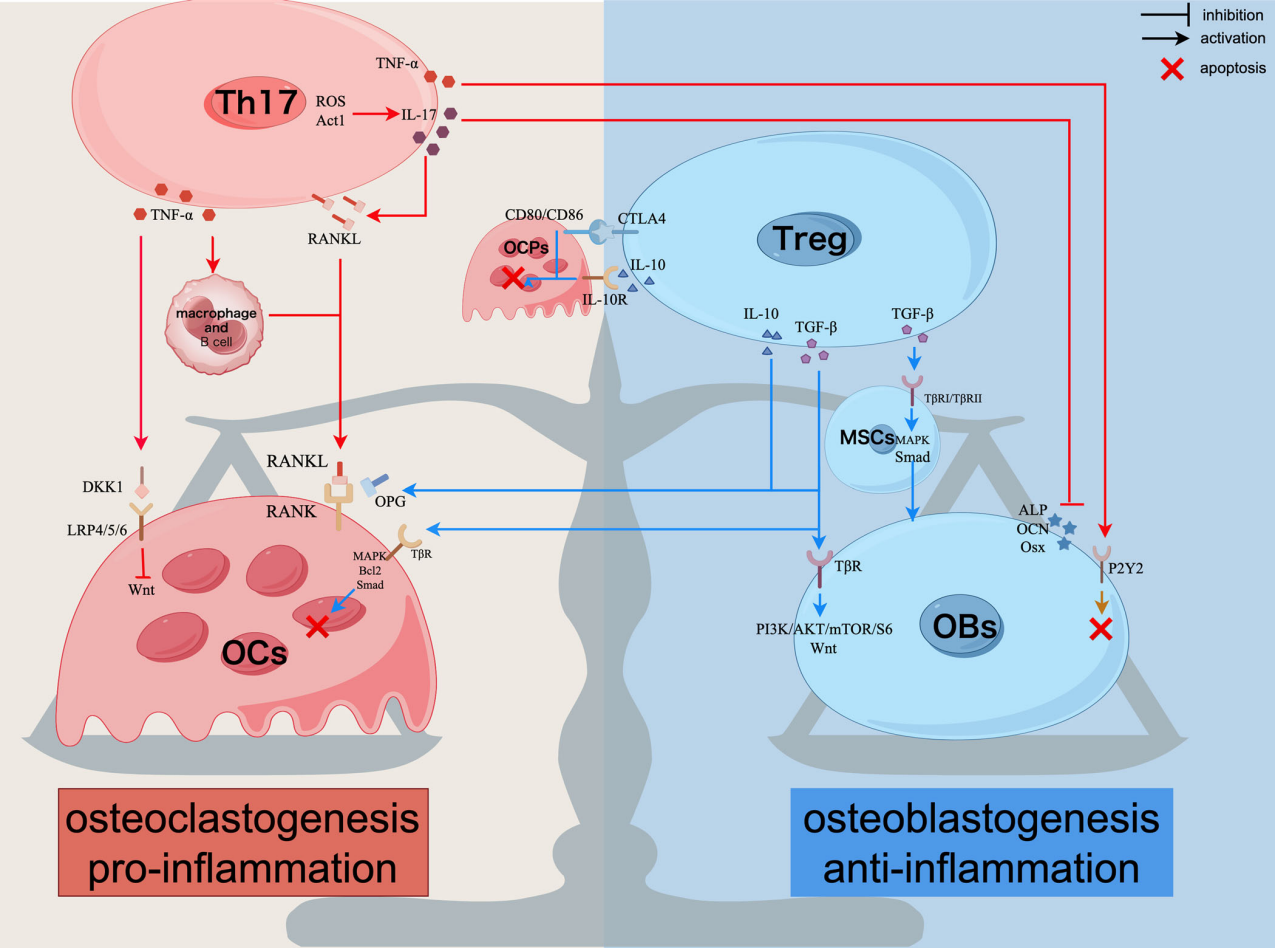
The balance between Th17 and Treg in osteoimmunology. In the intricate interplay of osteoimmunology, the scales of bone dynamics oscillate between the pro‐inflammatory implication of Th17 and the anti‐inflammatory influence of Treg. Th17 fosters osteoclastogenesis and bone resorption by emitting inflammatory cytokines, such as IL‐17 and TNF‐α, which mainly stimulate the RANKL/RANK pathway and suppress osteoblast activity. Conversely, Treg promotes bone formation by releasing cytokines such as IL‐10 and TGF‐β, activating osteoblast‐related signaling pathways including MAPK, Smad, Wnt, and PI3K/AKT/mTOR/S6. Additionally, Treg impedes osteoclast activity by inducing the apoptosis of their precursors and elevating OPG levels to inhibit RANKL from engaging with RANK. The delicate balance between Th17 and Treg determines the overall inflammatory milieu, which in turn dictates whether bone remodeling leans towards resorption or regeneration.

In summary, the influence of Th17/Treg on bone homeostasis is primarily manifested in the secretion of inflammatory cytokines by Th17 cells. These cytokines not only promote inflammatory responses but also enhance osteoclast differentiation and maturation. Simultaneously, Tregs inhibit inflammation, facilitate osteoblast proliferation and differentiation, and suppress osteoclast activity through the secretion of anti‐inflammatory cytokines and direct cell contact. The balance between Th17 and Treg in osteoimmunology is shown in Figure 1.

Over the immune system, a few additional functional reciprocal cell subtypes are collaborating with Th17/Treg to regulate bone homeostasis, including M1/M2 macrophage, Th1/Th2, Th22/Breg, γδ T/αβ T. The interplay between these cell balances occurs through a complex network involving cytokines, cell surface molecules, and signaling pathways,^37^ in preserving bone homeostasis as well as promoting bone regeneration.^95^, ^96^, ^97^ Meanwhile, activated lymphocytes interact and coordinate with innate immune cells to clean up the damage of infection and injuries then lead to health recovery.^98^

## 4 BONE TISSUE ENGINEERING BY MODULATING TH17/TREG FOR BONE REGENERATION

The main purpose of bone tissue engineering is to integrate the knowledge and technologies of biochemistry, materials science, and engineering to develop new strategies and materials to promote bone regeneration.

The imbalance of Th17/Treg activity is one of the important causes of aggravating bone injuries in rheumatoid arthritis, postmenopausal osteoporosis, periodontitis, and other diseases.^99^, ^100^ Recognizing the implication of Th17/Treg in osteoimmunology, more and more researchers have attempted to utilize bone tissue engineering to modulate Th17/Treg for bone regeneration.

Nano‐structures play a critical role in bone tissue engineering applications as they mimic the microenvironment and enable it to promote cell attachment, proliferation, differentiation, and new bone formation. Additionally, combined with 3D printing technology, the shape and structure of the scaffolds may be accurately controlled. These advantages have promoted the application of nano‐structures in bone tissue engineering.^8^, ^101^, ^102^

The ε‐poly‐l‐lysine‐coated nanoscale polycaprolactone/hydroxyapatite (EPL/PCL/HA) composite nano‐scaffolds developed by Bin Tian's team exhibited excellent biocompatibility.^103^, ^104^, ^105^ The team demonstrated that this nanocomposite scaffold could suppress pro‐inflammatory Th17, M1, and Th1 cells in rabbit bone defect models while facilitating the infiltration of anti‐inflammatory Th2 and Tregs. Moreover, it promoted the polarization and aggregation of M2 macrophages, thereby promoting new bone formation.^106^

Inhibiting Th17 and promoting Tregs have emerged as important therapeutic approaches for inflammatory bone injuries and autoimmune diseases.^99^ One treatment is combined immunosuppressive drug rapamycin with IL‐2 and TGF‐β.^107^ However, long‐term use of immunosuppressive drugs may lead to severe side effects.^108^ To achieve amplification and maintenance of Tregs while minimizing side effects, some researchers have turned their attention to microRNAs (miRNAs).

miRNA refers to a category of noncoding RNA strands that are typically about 22 nucleotides long. miRNAs target the 3′ untranslated region of mRNAs, then accelerating mRNAs degradation.^109^ In theory, if certain diseases are caused by abnormally high gene expression levels, it is possible to restore cell function and treat these diseases by negatively regulating the expression of these genes by specific miRNAs.^110^ Jeker et al. discovered the presence of miR‐10a in Tregs.^111^ Other researchers have incorporated IL‐2/TGF‐β and miR‐10a into nano‐sponge microspheres composed of materials such as poly (lactic acid)/polyethylene glycol/silicon dioxide and implanted them into mice to promote Treg‐mediated immunotherapy for bone loss.^112^ The nano‐structure itself has high miRNA affinity and negligible cytotoxicity enabling long‐term, continuous delivery of miRNA.^113^ Moreover, when delivering miRNAs through a scaffold, they are directly administered to the lesion instead of being dispersed via blood circulation. This targeted administration reduces toxicity towards organs like the liver or kidneys while minimizing interference from other tissues on miRNA. Consequently, smaller doses may be used to achieve therapeutic effects which further reduce potential side effects and improve patients’ tolerance to treatment.

The development of nano‐scaffold products typically involves a series of sophisticated techniques, including electrospinning, nanoparticle self‐assembly, microfluidic technology etc. The high costs of these techniques limited the transition of the nano‐structure from laboratory research to clinical applications. Hydrogels, like nano‐structures, are also commonly employed as scaffolds in bone tissue engineering.^114^, ^115^, ^116^ Hydrogels are generally more cost‐effective. Moreover, hydrogels offer advantages such as excellent biocompatibility, high water content, and the ability to mimic the extracellular matrix. When loading the hydrogel with 1,4‐dihydrophenonthrolin‐4‐one‐3‐carboxylic acid (1,4‐DPCA), a small molecule drug with angiogenic and bone‐forming properties by upregulating Vascular endothelial growth factor A (VEGF‐A) and Hypoxia‐inducible factor 1‐a (HIF‐1a), this hydrogel complex not only promotes CXCR4‐dependent Tregs accumulation but also facilitates alveolar bone regeneration in mice with experimental periodontitis‐induced bone loss.^117^, ^118^, ^119^ Although its application for treating human periodontitis has not been reported, locally injectable hydrogel containing 1,4‐DPCA has great potential as a convenient and practical therapeutic strategy.

Exosomes (EXOs) are extracellular vesicles secreted by cells and enable to delivery of proteins, RNA, and other signaling molecules to other cells, thereby facilitating intercellular communication.^120^, ^121^ In therapeutic applications, when free cytokines like TGF‐β1 and IL‐10 are administered to tissues, they are rapidly degraded, thus their effects to induce Tregs recruitment and to inhibit inflammatory bone loss are diminished, and the therapeutic efficacy is significantly reduced. Therefore, it is better to package these immunomodulatory factors (TGF‐β1, L‐10) into EXOs to protect them from protease degradation.^122^ The protection is crucial for these cytokines to exert their intended effects.

Researchers have isolated various subtypes of dendritic cell‐derived EXOs (DC‐EXOs) from Dendritic Cells (DCs) and then delivered the EXOs into the gingiva of mice with experimental periodontitis. When EXOs derived from Regulatory Dendritic Cells (regDCs) were used, the local DCs maturation and Th17 effector induction were inhibited and Tregs recruitment was promoted. Ultimately, this led to a restoration in bone loss.^122^ Possible mechanisms for these effects may involve the binding of EXOs to cell surface receptors, subsequently activating the downstream Smad2 signaling pathway. Alternatively, EXOs could be internalized by target cells after unloading their cargo. Another possibility is that TGF‐β1's slow release in the extracellular matrix promotes a paracrine effect on recipient cells.^123^, ^124^, ^125^, ^126^

The preliminary attempts mentioned above have yielded promising outcomes, indicating the potential value of EXOs in the protection of loaded cytokines and sustaining their long‐lasting effects. In current studies, MSCs serve as the primary source of EXOs regulating Th17/Treg. The research direction mainly focuses on treating periodontitis and suppressing alveolar bone absorption.^120^ EXOs derived from 3D cultured MSCs may restore Th17/Treg and resolve the immune imbalance in inflammatory periodontal tissues.^34^ The implication of MSC‐EXOs on T cells seems to be dose‐dependent.^127^ The underlying mechanisms are complex, involving regulatory networks such as iR‐1246/Nfat5 axis,^34^ Th17/Treg/miR‐155‐5p/SIRT1^128^ and NF‐κB.^129^

The immune system acts as a double‐edged sword. Other than defending infection and injuries, Immune cell imbalance may lead to excessive immune response, tissue damage, and autoimmune diseases.^99^, ^100^ Specifically, excessive activation of T cells may result in autoimmune diseases like rheumatoid arthritis and osteoporosis. Scholars have also made efforts in bone tissue engineering to address this excessive activation issue as well.^130^, ^131^

The balance between Th17 and Tregs plays a crucial role in establishing an immune microenvironment that facilitates bone regeneration. Numerous researchers have employed novel materials and innovative technologies to modulate this cell balance in the field of bone tissue engineering. Although most efforts are currently limited to in vitro experiments and animal models, significant progress has been made. As research advances and technology improves, it is anticipated that more effective treatment approaches will be developed to meet the clinical demand for bone injury management. The relevant technical strategies and mechanisms to modulate Th17/Treg for bonSe regeneration are summarized in Table 1.

**Table 1:** The relevant technical strategies and mechanisms to modulate Th17/Treg for bone regeneration.

Technical strategies	Models	Materials	Mechanisms	Outcomes	Ref
Nano‐structures	Mice	LDH‐CeO2	Neutralize hyperacidity to release Mg2+ and CeO2 NPs. The resultant ROS scavenging activity enhancement to polarize M1 into M2 via NF‐*κ*B pathway.	Accumulate Treg and inhibit Th17 and plasma cells.	[131]
	Mice	TDNs	Release MCP‐1 to induce the apoptosis of T cells through the conjugated FasL.	Skew Treg/Th17 cell balance.	[130]
	Mice	Nanofibrous Spongy Microspheres	MSNs and PLGA MS release miR‐10a as well as IL‐2/TGF‐β. PLLA NF‐SMS serves as a scaffold.	Treg enrichment, expansion against bone loss.	[112]
	Rabbits	EPL/PCL/HA nano‐scaffolds	Mimic the extracellular matrix and microenvironmental changes inside scaffold.	Promote Th2, M2 and suppress Th1, Th17, and M1.	[106]
Hydrogel	Mice	1,4‐DPCA hydrogel	Increasing HIF‐1α and VEGF‐A.	Promote CXCR4‐dependent accumulation of Treg.	[119]
Exosomes	Mice	EXOs from DCs	Bind to specific cell surface receptors, subsequently activate Smad2 pathway. EXOs could be internalized by target cells. TGF‐β1's gradual releasing facilitates a paracrine effect on recipient cells.	Protect TGF‐β1 and IL‐10 from degradation. Suppress maturation of DCs and induction of Th17 effectors. Promote Treg recruitment.	[122]
	Mice	EXOs from MSCs	Involve the miR‐1246/Nfat5 axis.	Restore Th17/Treg cell balance.	[34]
	Mice	EXOs from MSCs	The impact is dose‐dependent. The exact mechanism needs further exploration.	Increase Treg populations and decrease CD4+ and CD8+ T cells.	[127]
	Mice	EXOs from MSCs	Involve APCs not CD4+ T cells‐dependent pathway.	Promote Treg activity.	[132]
	In vitro	EXOs from periodontal ligament stem cells	Involve Th17/Treg/miR‐155‐5p/SIRT1 net.	Alleviate inflammatory microenvironment and keep Th17/Treg balance.	[128]
	Mice	EXOs from CD137‐modified endothelial cells	Involve NF‐КB pathway.	Promote Th17 differentiation.	[129]

## 5 SUMMARY AND PROSPECT

To summarize, the understanding of the immune system's role in bone homeostasis has significantly advanced. Primarily, innate immune cells need to decontaminate undesirable substances resulting from inflammation, fractures, tumors, implant surgery, and other factors. This process establishes a favorable microenvironment for bone regeneration. Subsequently, the immune system orchestrates various cellular activities to maintain a proper microenvironment over the entire bone remodeling process. Recognizing the key role of Th17/Treg balance in osteoimmunology has prompted scholars to explore novel materials and innovative technologies to modulate their cellular activity in bone tissue engineering for promoting bone regeneration.

In this review, the early role of the immune system is discussed about the complement system, neutrophils, and macrophages. These cells not only participate in phagocytosis and clearance but also affect bone‐related cells through various manners.^133^ When examining the mechanisms of Th17 and Tregs in maintaining a microenvironment conducive to bone regeneration, it becomes apparent that their effects on osteoclastogenesis and osteoblastogenesis are not all the way constant but rather influenced by factors such as timing of action, species types, and receptor types. Furthermore, crosstalk exists among different cytokines and signaling molecules; thus further clarification of these mechanisms is necessary.

## AUTHOR CONTRIBUTIONS

**Shuyu Zhu**: Conceptualization; resources; supervision; validation; visualization; writing—original draft; writing—review and editing. **Jing Zhou**: Conceptualization; supervision; writing—review and editing. **Zhigang Xie**: Conceptualization; funding acquisition; supervision; writing—review and editing.
